# Autonomy support and diastolic blood pressure: Long term effects and conflict navigation in romantic relationships

**DOI:** 10.1007/s11031-015-9526-6

**Published:** 2015-11-21

**Authors:** Netta Weinstein, Nicole Legate, Madoka Kumashiro, Richard M. Ryan

**Affiliations:** Cardiff University, Cardiff, UK; Department of Psychology, Illinois Institute of Technology, 3105 S. Dearborn St., Chicago, IL 60616 USA; Psychology Department, Goldsmiths, University of London New Cross, London SE14 6NW, UK; Institute for Positive Psychology and Education, Australian Catholic University, Strathfield, NSW Australia

**Keywords:** Autonomy support, Relationships, Diastolic blood pressure, Self-determination theory

## Abstract

Perceiving autonomy support—or encouragement to be oneself—from a romantic partner or other close relationship partners has been shown to yield a variety of psychological health benefits, but it is less clear how perceiving autonomy support from partners is linked to physical health. In two studies we examine the associations between receiving autonomy support in romantic relationships and diastolic blood pressure, an important indicator of cardiovascular health. Results of a longitudinal study found support for a model in which autonomy supportive romantic relationships are linked with lower diastolic blood pressure. Whereas Study 1 showed general longitudinal effects, Study 2 revealed the importance of receiving autonomy support from partners during times of conflict. Implications of the findings will be discussed in the context of self-determination theory.

## Introduction

Close relationships, and romantic relationships in particular, offer significant opportunities for promoting or undermining well-being and health. Indeed, a large body of evidence has linked highly supportive relationships with physical health (e.g., see Deci and Ryan 2008; Ng et al. 2012; Ryan et al. 2008). Although extant research also suggests that close relationships affect physical health (e.g., Berkman 1995; Cohen 1988; Robles et al. 2014; Uchino et al. 1996), the specific pathways through which relationships might influence health outcomes remain underexplored (e.g., Uchino 2009).

In the present research, we apply the framework of *self*-*determination theory* (SDT; Deci and Ryan 1985, 2014; Ryan and Deci 2000) to focus on an attribute of relationships that has been repeatedly associated with psychological well-being and relationship satisfaction: autonomy support (Deci et al. 2006; Weinstein et al. 2010; Weinstein 2014). We specifically examine connections between autonomy support from a romantic partner and diastolic blood pressure, an important indicator of physiological stress and cardiovascular health in younger and middle-age adults. Thus, the major aim of the present research was to examine previously unexplored associations between diastolic blood pressure and perceiving autonomy support from a partner, using longitudinal and lab methodologies.

## Autonomy support in close relationships

Self-determination theory conceptualizes *autonomy support* as supporting other individuals in their need for autonomy, or the need to act in accord with deeply held values and express oneself authentically (Ryan and Deci 2000). People have a universal need to experience autonomy, and others play a key role in supporting the autonomy of those around them (La Guardia and Patrick 2008). When individuals are being supported in their autonomy needs, they are encouraged to express and pursue their choices and values and experience a relatively low amount of pressure and coercion.

Though other relationships also provide autonomy support, autonomy-supportive romantic partners are likely to be particularly influential due to the highly interdependent nature of romantic relationships, which also exert regular influence across a variety of different situations (e.g., Kelley 1983). Autonomy-supportive partners allow for self-expression—freely and without fear of judgment or retribution—and offer respect for their partner’s values and interests (La Guardia and Patrick 2008). When experiencing autonomy support from a romantic partner, love is felt as unconditional—individuals feel loved in being who they are, rather than only feeling loved when partners’ desired behaviors or expectations are met (Assor et al. 2004; Knee et al. 2008).

## Benefits of autonomy support

People benefit in myriad ways when they perceive autonomy support from others, and particularly from close relationship figures like parents, best friends, and romantic partners (e.g., La Guardia et al. 2000; Ryan et al. 2015). Autonomy support has been linked to a variety of positive relationship outcomes including emotional reliance (Ryan et al. 2005), honest interactions with close others (Hodgins et al. 1996), and more relationship satisfaction and commitment (Blais et al. 1990; Patrick et al. 2007; Patrick et al. 2009).

Along with their interpersonal benefits, autonomy-supportive romantic relationships can foster a sense of well-being. For example, perceiving autonomy support from a partner predicts greater self-esteem, vitality and positive affect, and less depression (e.g., Custers et al. 2010; Legate et al. 2012; Patrick et al. 2007). More relevant for the present work, perceiving autonomy support from a partner is related to increased engagement in health behaviors. For example, studies show that perceiving autonomy support from close partners (most of whom were romantic partners) is linked to more physical activity and healthy eating through its effects on health motivation (Ng et al. 2014) and to long-term weight loss outcomes among individuals participating in a weight-loss trial (Gorin et al. 2014).

Outside of the domain of romantic relationships, some recent research has begun to link autonomy support to stress responses (for a review see Weinstein and Ryan 2011)—an important pathway through which autonomy support may impact varied health outcomes (e.g., Thompson and Prottas 2006). Early studies using physiological indicators of stress have shown some support for these ideas. For example, athletes with less autonomy-supportive coaches showed higher salivary immunoglobulin A prior to practice sessions, an indicator of acute stress (Bartholomew et al. 2011). Though not assessing autonomy support directly, Quested et al. (2011) found that dancers with high satisfaction in their need for autonomy—a likely consequence of autonomy-supportive environments—had lower cortisol responses following a performance than those with low need satisfaction. These early studies point to a link between autonomy support and physiological measures of stress, but we are unaware of any research directly linking autonomy support in close relationships, and romantic partners in particular, to physiological indices of stress.

In this research, we focus on whether autonomy support experienced within romantic relationships affects blood pressure, an indicator of stress with long-term implications for cardiovascular disease (see Robles and Kiecolt-Glaser 2003 for a review). We speculate that romantic relationships might be important in shaping blood pressure in part because of the increased opportunities for conflict, which exists in almost all romantic relationships (Canary et al. 1995), and because people experience conflict differently as a function of the level of autonomy support they perceive from romantic partners (Knee et al. 2005).

## Autonomy support during conflict

Evidence suggests that autonomy-supportive individuals continue to trust and seek to understand a partner’s perspective even during periods of conflict, which then benefits the relationship. For example, La Guardia and Ryan (2007) showed that autonomy-supportive partners tended to have more emotional awareness and openness in processing their emotions. Behaving in these relationship-maintaining ways, such as continuing to provide support and understanding during a conflict, in turn leads to fewer negative emotions following conflict (Knee et al. 2002).

Furthermore, research more generally shows a reliable association between marital conflict and heightened blood pressure (see Robles and Kiecolt-Glaser 2003 for a review), and hostile conflict behaviors such as directly invalidating a partner’s feelings have been shown to have particularly deleterious effects on blood pressure (Ewart et al. 1991). It should follow, then, that autonomy-supportive behaviors during conflict, such as supporting partners’ feelings and seeing things from their perspective, might help protect against rises in blood pressure even in these tense situations. We these expect that the benefits of perceiving autonomy support from a partner will manifest beyond self-reported health and well-being reactions. Based on previous work in romantic relationships, we believe that higher global perceived autonomy support will be linked to recalling a partner’s behaviors during conflict as more autonomy supportive, which will in turn link to lower levels of blood pressure.

## Diastolic blood pressure

This research focuses on blood pressure both because it has important effects on cardiovascular health and because past studies have suggested that it is closely tied to psychosocial stressors (e.g., Rainforth et al. 2007). We specifically focus on diastolic blood pressure because it is the predominant indicator of physical concerns in younger adults (Franklin et al. 2001; Kannel et al. 1971). Furthermore, medical interventions with younger samples show effects on diastolic but not systolic blood pressure (Grobbee and Hofman 1986). In turn, diastolic blood pressure is a robust indicator of cardiovascular health; for example, large community samples indicate it is most closely associated with relative artery wall thickness (Devereux et al. 1983; Schnall and Pieper 1990), and that reductions in diastolic blood pressure may result in decreases in the prevalence of hypertension and stroke (Cook et al. 1995). Randomized controlled studies show that intensive diastolic blood pressure control (maintaining blood pressure around 75 mmHg) decreases mortality in people with diabetes (Estacio et al. 2000). Moreover, in the general population, diastolic blood pressure was shown to be the strongest predictor of coronary heart disease and of cardiovascular risk in a sample of young adults (Franklin et al. 2001; Raitakari et al. 1994).

In particular, psychosocial stress has powerful effects on diastolic blood pressure (c.f., meta-analyses by Rainforth et al. 2007; Uchino et al. 1996). More importantly for the current research, romantic relationships may be especially linked to blood pressure. One meta-analysis indicates a robust relation between social support, broadly defined, and diastolic blood pressure (Thorsteinsson and James 1999). Cross-sectional work in the community has shown that people who are unhappy in their marriage have higher blood pressure than single people, even if they have a supportive social network outside of the relationship (Holt-Lunstad et al. 2008). In addition, support from a partner, such as expressed by a supportive touch, is linked with lower blood pressure (e.g., Light et al. 2004). A recent meta-analysis (Robles et al. 2014) also found that being in a marriage high in satisfaction and low in hostility is linked to lower cardiovascular reactivity, including diastolic blood pressure, during conflict. Taken together, it is clear that support from a partner impacts blood pressure, but the current work focuses on a specific type of support—autonomy support—and explores one way through which autonomy support benefits health.

## Research overview

In two studies we examine the effects of perceiving autonomy support from a romantic partner on diastolic blood pressure. Study 1 involved a 2-year long longitudinal study to examine trajectories in blood pressure as a function of perceived autonomy support from romantic partners. In Study 1 we hypothesized that recipients of autonomy support would show lower levels of blood pressure over time when compared to those who did not feel supported in their autonomy (H1).

Study 2 asked participants in the lab to reflect on a conflict with partners in order to examine the role of conflict in linking global autonomy support and blood pressure. Based on the research discussed above we expected that recalling autonomy-supportive behaviors during conflict would explain some of the variability shared between global perceived autonomy support and changes in blood pressure. Specifically, we hypothesized that:

### H2A:

Global perceived autonomy support from one’s partner would be linked to lower blood pressure after reflecting on a conflict with one’s partner.

### H2B:

Recalling autonomy-supportive behaviors *duringconflict* would be linked to lower blood pressure after conflict and would mediate links with global autonomy support.

In both studies, we controlled for the potential effects of attachment style as insecure attachment negatively affects responses to interpersonal conflicts (Carpenter and Kirkpatrick 2005; Feeney and Kirkpatrick 1996; Gallo and Matthews 2006; Gouin et al. 2009; Mikulincer and Shaver 2007) and has been linked to autonomy support in past research (La Guardia et al. 2000). In addition, in Study 1 we controlled for relationship commitment to reflect relationship quality (Wong and Sohal 2002) and physical symptoms as an indicator of physical health that might be responsible for the links between autonomy support and blood pressure (Pennebaker et al. 1982).

## Study 1

In Study 1 we sought to examine long-term (2-year) changes in diastolic blood pressure as a function of perceiving autonomy support from romantic relationship partners. Assessing changes across time allows us to control for the high level of natural variability between individuals’ blood pressure (Obrist et al. 1978) and holds more implications for long-term health (MacMahon et al. 1990). We were specifically interested in perceptions of receiving autonomy support; therefore, Partner A’s (from now on will be called *support provider*) own report on providing autonomy support to partner B (who we will call *recipient*) was controlled for to account for the possibility that being in autonomy-supportive relationships in general, rather than receiving autonomy support, specifically, accounted for changes in blood pressure (Deci et al. 2006). In addition, we controlled for other factors that might influence the association between receiving autonomy support and blood pressure, including insecure attachment styles (e.g., Mikulincer and Shaver 2007), relationship commitment as an indicator of relationship quality that might shape partners’ wellness (Dush and Amato 2005), and physical symptoms as a proxy for health that might influence blood pressure (Pennebaker et al. 1982). We hypothesized that receipt of autonomy support would predict trajectories of blood pressure across the 2 years: those who experienced more autonomy support from their partner would have lower blood pressure across time relative to those who experienced less autonomy support (H1).

## Participants and procedure

Participants were both partners of a romantic couple who reported on perceived partner autonomy support and blood pressure on three occasions during a 2-year longitudinal study (a lag of 1-year between measurement points for this study). At the start of the study, 187 couples took part in the project (183 heterosexual couples, 4 lesbian couples), with the number of couples dropping to 139 and 98 at the respective subsequent readings (1 year in and 2 years in). The number of completed sessions correlated with perceived partner autonomy support, *r* = .13, *p* = .02, although it did not correlate with blood pressure, *r* = −.02, *p* = .75. HLM analysis used with the data is better equipped to handle these missing cases than multivariate regression (Little and Rubin 2014). Participants were 26.47 years old on average, and mostly Caucasian (86 %). There was a good distribution among dating, engaged, and married couples (25 % dating steadily, 29 % engaged, 38 % married, 8 % other), with a majority of the couples living together (84 %).

## Materials

### Perceived partner autonomy support

During Time 1, at the start of the study, both individuals reported on the extent to which their partner provided autonomy support. Perceived autonomy support from one’s partner was measured with three items adapted from La Guardia et al. (2000). Items including “When I am with my partner, I have a say in what happens, and I can voice my opinion” were paired with a scale ranging from 0 (*do not agree at all*) to 8 (*agree completely;* α = .71).

### Attachment style

The Experiences in Close Relationships-Revised (ECR-R) Questionnaire (Fraley et al. 2000) measured avoidant and anxious attachment styles at Time 1. This 36-item scale includes items such as “I am nervous when partners get too close to me” (avoidant attachment) and “I often worry that my partner doesn’t really love me” (anxious attachment) using a scale ranging from 0 (*do not agree at all*) to 8 (*agree completely*). Reliability across all items was high, α = .94, and so the items were averaged to create an overall insecure attachment score, similar to those used by La Guardia et al. (2000).

### Commitment

Relationship commitment at Time 1 was assessed using a 15-item scale (Rusbult et al. 2009) that measured intent to persist in the relationship, long-term orientation, and psychological attachment (e.g., “I am completely committed to maintaining our marriage”; 0 = *do not agree at all*; 8 = *agree completely*; α = .89).

### Physical symptoms

Physical health was controlled for at all three time points using a modified version of the Choen-Hoberman Inventory of Physical Symptoms (CHIPS), which is a 33-item scale that asks participants to check yes if they experienced each of the physical health symptoms over the last 6 years (e.g., “sleep problems,” “cold or cough”; *Yes* = *I Have Had This Problem During The Past Six Months*). The ‘yes’ responses were given a score of 1, and the 33 items were summed to create an overall physical symptoms index (αs = .82, 82, and 80 for the three time-points).

### Blood pressure

Blood pressure was assessed noninvasively at all three time-points by study experimenters, using an automatic oscillometric wrist cuff placed on the left hand of seated participants. Measurements were taken 20 min to 1.5 h after the start of the lab session at each time-point. Participants were seated and the monitor was placed close to heart level. Both diastolic and systolic measures were collected; diastolic blood pressure measures the pressure in blood vessels when the heart rests between beats, whereas systolic blood pressure measures the pressure in the blood vessels when the heart beats (for more information, see Stamler 1991).

## Results

Data were screened to ensure they were homoscedastic and normally distributed. Kurtosis and skewness were at least at marginally acceptable levels for all measures (skewness <1.4; kurtosis <1.9), but because diastolic blood pressure and partner autonomy support showed somewhat non-normal distributions (partner autonomy support = 1.89, diastolic blood pressure = 1.37) we analyzed models using log-transformed data; findings were consistent across models using both raw and transformed data.

The data were non-independent across both longitudinal measurements and within couples. Given this, the general analytic strategy involved a two-level multilevel model in which effects for each of the two partners were initially modeled separately at Level 1 (Bolger and Laurenceau 2013; Kenny et al. 2006; Raudenbush et al. 1995). All models allowed error variances to differ across partners and also allowed residual variance to correlate between partners within each couple. We then pooled coefficients that did not differ across partners.

Our model tested physical symptoms (as a proxy for health) at Level 1. At Level 2 we also tested the main effects of gender on blood pressure, recipient’s and support provider’s commitment at Time 1, recipient’s attachment style at Time 1, and recipient’s and support provider’s perceived autonomy support at Time 1. Furthermore, at Level 2 we tested for moderation of time by: recipient’s and support provider’s commitment, recipient’s attachment style, and recipient’s and support provider’s autonomy support. All variables except for gender were grand-centered per recommendations by Bryk and Raudenbush (1992).

### Gender variability

Tests of model fit examined whether partners were distinguishable by constraining an effect to be equal across both spouses and examining change in model fit (Ackerman et al. 2011). It is sensible to pool spouses if the hypothesized links do not vary across husbands and wives. A model tested the relative slopes for time X perceived autonomy support (as part of the full model) separately for both men and women excluding the four lesbian couples in this case. A contrast comparing these two groups showed no difference in the relation between perceived autonomy support on time X recipients’ perceived autonomy support for these two groups: χ^2^ = .161, *p* > .50; systolic: χ^2^ = .758, *p* > .50. A second model including the four lesbian couples found nearly identical results; χ^2^ = .162, *p* > .50; systolic: χ^2^ = .753, *p* > .50. As such, the model was pooled across both partners of the couple and included the lesbian couples.

### Diastolic blood pressure

**Fig. 1:**
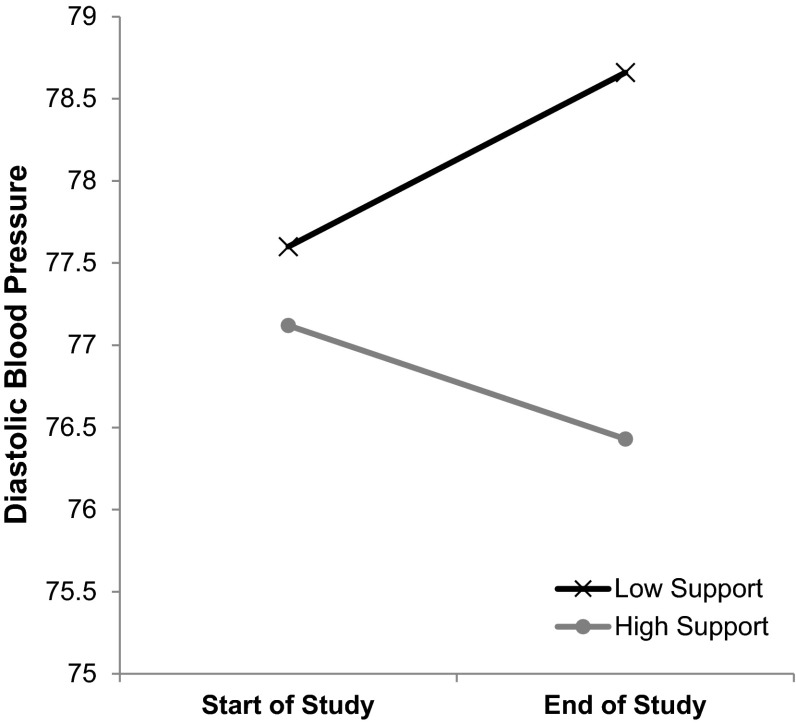
Study 1 interaction between perceived partner autonomy support and time in predicting blood pressure across 2 years

As shown in Table 1, there were two significant main effects of gender on blood pressure, with women demonstrating lower blood pressure than men across the 2 years, *b* = −0.012, *t*(348) = −2.41, *p* = .02, *d* = .08, and blood pressure at baseline showing significant positive associations to blood pressure across the 2 years, *b* = 0.004, *t*(348) = 16.93, *p* < .001, *d* = 1.90. None of the other Level 2 main effects were significant, *ts* < 1.60, *ps* > .10 (see Table 1 for the full results). At Level 1, overall there was no change in blood pressure across the 2 years as this main effect of time was non-significant, *b* = −0.000, *t*(328) = −0.07, *p* = .95, *d* = .01. Recipients’ insecure attachment did not influence this trajectory, *b* = −0.00, *t*(328) = −1.59, *p* = .11, *d* = .17. Recipients’ commitment was marginally linked to a relative decrease in blood pressure over time (showing a marginal interaction effect), *b* = −0.003, *t*(328) = −1.97, *p* = .05, *d* = .22, though there was no interaction with support providers’ commitment, *b* = 0.001, *t*(328) = 0.54, *p* = .59, *d* = .06. Although the support providers’ autonomy support (presumably reflecting one’s *giving* of support) did not moderate this relation, *b* = 0.001, *t*(328) = 0.87, *p* = .39, *d* = .10, recipients’ perceived autonomy support from his or her partner (presumably, one’s *receiving* of support) interacted with time, *b* = −0.004, *t*(328) = −2.35, *p* = .02, *d* = .34. Follow-up analyses (Aiken et al. 1991) showed moderate decreases in blood pressure across 2 years for those who perceived high levels of autonomy support (+1*SD*) from their partners, *b* = −0.57, *t* = −2.87, *p* = .005, *d* = .45, whereas those who initially perceived their partners to be low in autonomy support (−1*SD*) demonstrated moderately higher blood pressure across the 2-year period, *b* = 1.00 *t* = 2.74, *p* = .007, *d* = .43 (see Fig. 1). In a second model, attachment style did not moderate the main effect of perceived autonomy support, *b* = −0.000, *t*(347) = −0.13, *p* = .90, *d* = .02, and did not further moderate the interaction between attachment style and perceived autonomy support, *b* = 0.000, *t*(327) = 0.36, *p* = .72, *d* = .04.

**Table 1:** Results of main models for diastolic and systolic blood pressure across both studies

	Diastolic		Systolic	
	*t*	*d*	*t*	*d*
*Study 1*				
Gender	2.02*	.08	−7.90**	.87
Physical symptoms	1.52	.22	0.10	.01
Insecure attachment	−1.51	.16	−1.43	.16
Own commitment	−0.46	.05	−0.34	.04
Partner commitment	0.40	.04	−0.40	.04
Own autonomy support	−0.13	.02	0.59	.07
Partner autonomy support	1.40	.15	−0.36	.04
Time	−0.07	.01	−3.59**	.40
Time X insecure attachment	−1.56	.17	−0.93	.10
Time X own commitment	−1.97	.22	0.96	.11
Time X partner commitment	0.54	.06	−0.62	.07
Time X own perceived support	−3.04**	.34	−0.17	.02
Time X partner perceived support	0.87	.10	−1.13	.13
*Study 2*				
Gender	−0.14	.05	−1.22	.40
Blood pressure baseline	11.78**	3.87	10.83**	3.56
Attachment style	1.12	.37	−0.59	.20
Perceived support	−2.10*	.69	−0.24	.08

Table presents all predictors tested in HLM (Study 1) models and linear multiple regressions (Study 2)

* *p* < .05; ** *p* < .01

### Systolic blood pressure

Findings indicated a main effect for gender, in that women had lower systolic blood pressure than men across the 2 years, *b* = −0.032, *t*(348) = −7.90, *p* < .001, *d* = .87. A main effect was also found for systolic blood pressure at baseline, which was linked to higher systolic blood pressure at Level 1, *b* = 0.000, *t*(348) = 12.13, *p* < .001, *d* = 1.34. There were no other significant main effects, *t*s < −1.43, *ps* > .15 (see all results in Table 1). Systolic blood pressure decreased across 2 years of the study, *b* = −0.003, *t*(328) = −3.59, *p* < .001, *d* = .40. Time was not moderated by recipients’ perceived autonomy support, *b* = 0.000, *t*(328) = −0.17, *p* = .87, *d* = .02, support providers’ perceived autonomy support, *b* = −0.001, *t*(328) = −0.13, *p* = .26, *d* = .13, recipients’ insecure attachment, *b* = −0.001, *t*(685) = −0.93, *p* = .35, *d* = .10, recipients’ commitment, *b* = 0.001, *t*(328) = 0.96, *p* = .34, *d* = .11, or providers’ commitment, *b* = −0.001, *t*(328) = 0.62, *p* = .54, *d* = .07. Attachment style did not moderate the main effect of perceived autonomy support, *b* = 0.001, *t*(347) = 1.22, *p* = .23, *d* = .13, and did not further moderate the interaction between attachment style and perceived autonomy support, *b* = 0.002, *t*(327) = 1.18, *p* = .24, *d* = .13

## Discussion

Study 1 supported the hypothesized relation between perceived partner autonomy support and diastolic blood pressure over time (our H1 above), though there were no relations with systolic blood pressure. Results showed that individuals who perceived their romantic partners to be autonomy supportive showed significant decreases in diastolic blood pressure across 2 years, whereas those who perceived their partners to be less supportive (less than the mean level of support) significantly increased in diastolic blood pressure across a 2-year period. Effect sizes for these changes in diastolic blood pressure as a function of perceived autonomy support were moderate. Moreover, this effect seemed to be independent of gender, attachment style, commitment to the relationship, and physical health. It appears to be that the autonomy support perceived from one’s partner that matters; one’s own provision of autonomy support did not show an impact on blood pressure trajectories.

Findings from this study led to the question: what is it about autonomy-supportive partners that relates to lower diastolic blood pressure? Notably, diastolic blood pressure in younger people is most affected by situations that are low or high in stress (Rainforth et al. 2007). In romantic relationships, where stressful conflict is almost always inevitable (Canary et al. 1995), perceptions of autonomy support and the way partners behave during conflict (Robles and Kiecolt-Glaser 2003) may be especially likely to affect blood pressure. Study 2 was designed to explore these ideas.

## Study 2

In Study 2, we examine a particular component of autonomy support that may help explain the effects identified in Study 1: context-specific perceptions of how autonomy supportive one’s partner is during conflict. To this end, participants were asked to recall a meaningful conflict with their romantic partners, and we examined change in blood pressure before and after this task as a function of initial reports of *global* perceived partner autonomy support, expanding on the measurement from Study 1. We also examined whether recalling a partner’s behaviors as autonomy supportive *during the conflict* might be responsible for effects on blood pressure, again controlling for attachment style. Finally, in this study we included a more robust measurement of global levels of autonomy support by assessing both perceived partner support for autonomy—a positive indicator of support—and perceived partner conditional regard—a negative indicator of support. Although autonomy-supportive partners allow for both satisfaction of needs (e.g., self-expression without fear of judgment or retribution; La Guardia and Patrick 2008) and lack of deprivation of needs (e.g., conveying that love is not conditional on approval or disapproval of partner’s actions; Assor et al. 2004; Knee et al. 2008), global autonomy support in Study 1 only assessed the positive component. This multidimensional approach acknowledged new trends to explore both the satisfaction and deprivation of needs, which both contribute to variance in well-being (Deci and Ryan 2014).

We hypothesized that individuals who perceived their partners to be more globally autonomy supportive and less conditionally regarding would show lower blood pressure after reflecting on a conflict with their partners (H2a), relative to those who perceived their partners to be less supportive in these ways. We further hypothesized that recalling partners as autonomy supportive during the conflict would mediate this effect (H2b).

### Participants and procedure

Forty-two participants who were in a romantic relationship took part in the study, with ages ranging from 19 to 74 (*M* = 28.22, *SD* = 13.64). Only one member of the couple participated in this study. Of participants, 22 were women, and 20 were U.K. nationals (participants were also from Cyprus, Germany, India, Romania, Slovakia, among other countries). Seven participants were married, and participants were in the relationship for an average of 67.61 months (SD = 137.71 months), or 5.63 years. The number of months in a relationship did not relate to changes in blood pressure across the study, *r* = −.05, *p* = .78.

Participants completed an initial survey assessing both perceptions of global partner autonomy support and their own attachment style. Following the survey, blood pressure was measured using an arm cuff twice in a row, to reduce likelihood of measurement error for this baseline measurement. All participants were then given the following simple instructions to think about a recent conflict with their partner: “*For the next 8* *min, please discuss an important conflict that you have had in the last 6* *months. Please think of a conflict that hasn’t yet been resolved*”. These instructions were intended to make salient a conflict with their partners and to elicit stronger emotions by asking participants to think back to an unresolved conflict (Friedman et al. 2000). They were then left alone in the lab for 8 min while they audio recorded their response to the request. Following this task, participants were asked to respond to a short survey assessing recalled partner support during the conflict and a second set of blood pressure measurements was taken.^1^ Participants were fully debriefed on the nature of the study before leaving.

### Materials

#### Blood pressure

The acquisition hardware used was an automatic oscillometric wrist cuff placed on the left hand of seated participants. Diastolic blood pressure ranged from 51 to 106 at the start of the study and 50–108 following the manipulation. Correlations between the two measurement points at baseline and post-conflict were *r* = .88 and .90, respectively. The two measurements were averaged at each time point; measurements at the start and end of the study correlated, *r* = .89.^2^

#### Global perceived partner autonomy support

For this study, we measured perceptions of partner autonomy support with the three items used in Study 1 (La Guardia et al. 2000), as well as perceptions of negative conditional regard from one’s partner, a form of control. Relationship-specific negative conditional regard was assessed with five items adapted from the Conditional Regard Index (Assor et al. 1997; Roth et al. 2009). Directions asked participants to: “Think back to your partner. How does your partner react when you do something he/she doesn’t like?” and included items such as “expresses less warmth toward me than usual”. All items were paired with a five-point scale ranging from 1 (*not at all true*) to 5 (*completely true*). The two scales were averaged (after negative items were reversed) to create a composite of partner autonomy support, with higher scores on the overall measure reflecting more autonomy support. Alpha for all items was acceptable, α = .77.

#### Recalled autonomy support during conflict

Recalled autonomy support during conflict was measured with five items adopted from the perceived autonomy support climate questionnaires (Black and Deci 2000; Mageau et al. 2015) including: “During this conflict, my partner understood me,” “During this conflict, my partner really listened to me,” and “During this conflict, my partner tried to see things from my point of view”. Items were paired with a scale ranging from 1 (*not at all true*) to 7 (*extremely true*). Reliability for the five averaged items (none were reversed) was α = .94.

### Attachment style

Attachment style was measured using the four-item Relationship Questionnaire (RQ; Bartholomew and Horowitz 1991); this shorter scale is a widely used proxy for the longer scales (Cassidy 2002) and was intended to reduce participant burden. Participants selected one item which best described them from four options representing insecure (3 items) or secure (1 item) attachment styles. Secure attachment was assessed with the item: “It is easy for me to become emotionally close to others. I am comfortable depending on them and having them depend on me. I don’t worry about being alone or having others not accept me”. Secure attachment was contrasted with insecure attachment styles, including: “I am uncomfortable getting close to others. I want emotionally close relationships, but I find it difficult to trust others completely, or to depend on them. I worry that I will be hurt if I allow myself to become too close to others”. Seventeen participants were classified as insecurely attached, and 24 classified as securely attached; participants received a score of ‘1’ if showing an insecure (anxious, avoidant, or ambivalent) style and ‘−1’ if secure. The approach of contrasting secure with insecure attachment styles is in line with the categorical nature of this scale and previous work employing the measure (see Cassidy 2002; Griffin and Bartholomew 1994).

## Results

### Analytic strategy

Data were screened to ensure they were homoscedastic and normally distributed (kurtosis and skewness <.90 for all measures). Hierarchical regression analyses predicted the criterion variables—post-task blood pressure and recalled partner autonomy support during conflict—from covariates, namely: baseline blood pressure (when predicting post-task blood pressure only), gender, and attachment style at Step 1, global perceived partner autonomy support at Step 2, and two-way interactions between attachment and global partner autonomy support at Step 3. Mediation analyses were conducted using the Process procedure (Hayes 2013) to obtain bias-corrected bootstrapped estimates based on 10,000 bootstrapping samples.

### Diastolic blood pressure

**Fig. 2:**
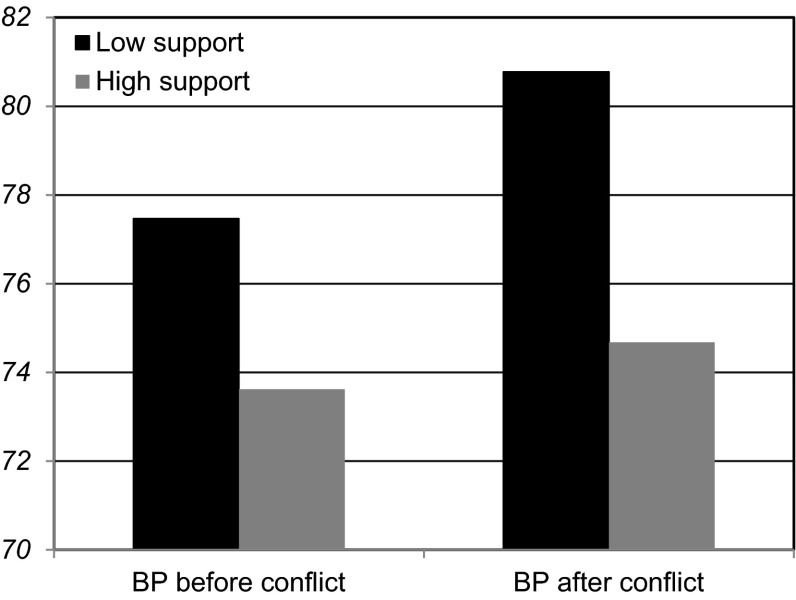
Study 2 diastolic blood pressure before and after the task of reflecting back to a conflict with one’s partner as a function of global perceived autonomy support from partner

At Step 1, baseline diastolic blood pressure was linked to blood pressure after the task, β = .90, *t*(37) = 11.78, *p* < .001, *d* = 3.87, though there were no associations with gender, β = −.01, *t*(37) = −0.14, *p* = .89, *d* = .05 or attachment style, β = -.08, *t*(37) = 1.12, *p* = .27, *d* = .37. At Step 2, global perceived partner autonomy support related to lower diastolic blood pressure, β = −.15, *t*(36) = −2.10, *p* = .04, *d* = .69 (this moderate effect supported our hypothesis H2a; see Fig. 2). At Step 3, global perceived partner autonomy support and attachment style did not interact, β = .03, *t*(35) = 0.16, *p* = .88, *d* = .05.

### Systolic blood pressure

At Step 1, baseline systolic blood pressure was linked to systolic blood pressure after the task, β = .86, *t*(37) = 10.83, *p* < .001, *d* = 3.56, though there were no associations with gender, β = −.01, *t*(37) = −1.22, *p* = .23, *d* = .40 or attachment style, β = .04, *t*(37) = 0.59, *p* = .56, *d* = .20. At Step 2, there was no link between global autonomy support and systolic blood pressure, β = −.02, *t*(36) = −0.24, *p* = .82, *d* = .08. At Step 3, global perceived autonomy support and attachment style did not interact, β = .17, *t*(35) = 0.69, *p* = .50, *d* = .23.

### Recalled partner autonomy support during conflict

At Step 1, neither gender, β = −.21, *t*(38) = −1.30, *p* = .20, *d* = .42, nor attachment style, β = .15, *t*(38) = 0.97, *p* = .34, *d* = .31, were associated with recalled autonomy support during conflict. At Step 2, global perceived partner autonomy support moderately related to recalled autonomy support during the conflict, β = .33, *t*(37) = 2.18, *p* = .04, *d* = .71. At Step 3, there was no interaction between global perceived autonomy support and attachment style in predicting recalled autonomy support during conflict, β = −.15, *t*(35) = 0.42, *p* = .68, *d* = .14.

### Mediation model for diastolic blood pressure

We expected that recalled autonomy support during conflict would mediate the association between global perceived autonomy support and blood pressure. To test this, in a final model, global perceptions of partner autonomy support was included as a predictor in Step 1 of the model, and findings showed that those who recalled more support from their partners during the conflict (our proposed mediator) had lower diastolic blood pressure following the conflict, controlling for baseline blood pressure, β = −.15, *t*(37) = −2.09, *p* = .04, *d* = .69. When taking this moderate effect of recalled autonomy support during conflict into account, the effect of global perceived partner autonomy support fell to non-significance, β = −.10, *t*(36) = −1.31, *p* = .20, *d* = .44, suggesting the presence of mediation. This mediation hypothesis was tested using the Process procedure (Hayes 2013) to obtain bias-corrected bootstrapped estimates based on 10,000 bootstrapping samples. These analyses indicated the indirect effect of recalled autonomy support during conflict was significant, data = 1.174, SE = .784, CI = −3.2981 to −.0062, supporting our hypothesis H2b. In other words, global partner autonomy support seemed to help lower diastolic blood pressure at least in part because the partner is recalled as being autonomy supportive even in the midst of conflict.

## Discussion

Study 2 supported findings from Study 1 showing potential benefits of perceiving one’s partner as being autonomy supportive on blood pressure, and identified one factor that may contribute to this relation. Specifically, mediation analyses indicated that recalling autonomy support from a partner during a conflict may partially buffer the harmful effects of the conflict on blood pressure (e.g., Robles and Kiecolt-Glaser 2003). Interestingly, this relation was independent of both gender and attachment style, and did not interact with attachment style. That is, individuals showed higher blood pressure after reflecting on a conflict with a non-supportive partner regardless of whether their attachment style was secure or insecure.

## General discussion

Based on previous research in self-determination theory, across two studies we tested whether autonomy-supportive romantic partners would positively impact health, specifically by reducing or keeping blood pressure low. Results of the first study supported our hypothesis over time (2 years), and a second study highlighted the importance of recalling autonomy support during conflict for lower blood pressure.

Study 1 utilized a longitudinal design to show the long-term health relations of being with an autonomy-supportive romantic partner. Specifically, findings indicated that blood pressure dropped over time among people who perceived their partners to support their autonomy needs, whereas blood pressure rose over time for people who experienced their partners as low in providing autonomy support. This is consistent with the high importance of romantic partners to wellness (La Guardia et al. 2000), but offers a new, physiological dimension, to understanding well-being outcomes of autonomy support in close relationships. Further, though there is a consensus that ‘good’ marriages protect cardiovascular health (e.g., Johnson et al 2000; Robles et al. 2014), most research on romantic relationships only reveals the costs of unsupportive, bad relationships on cardiovascular health (e.g., Orth-Gomer et al. 2000). This is one of the few studies demonstrating possible gains in cardiovascular health over time as a function of being in a supportive relationship, and to our knowledge, the first to look specifically at blood pressure.

A laboratory study then tested the role of autonomy support by having people think back to an unresolved conflict with their partners while measuring changes in blood pressure. Results showed that the pattern of change was predicted by the autonomy supportiveness of these romantic relationships. The findings supported the hypothesized positive effect of autonomy-supportive partners on blood pressure. Both Study 1 and 2 results for autonomy support were independent of attachment style, which is important because a literature shows a link between having a secure attachment style and less physiological change to social conflict (Gallo and Matthews 2006), and a separate literature suggests that those who are insecurely attached may also perceive their partners as less autonomy supportive (La Guardia et al. 2000). Moreover, the absence of an interaction with attachment highlights the importance of autonomy support regardless of attachment style; in other words, a secure attachment may not protect someone from the harmful physiological effects of being in a non-supportive relationship, and an insecure attachment would not necessarily obscure the benefits of being with an autonomy-supportive partner.

Although we are suggesting that partner autonomy support can have beneficial effects on blood pressure, neither of these studies manipulated partner autonomy support directly. This was intentional, as we were interested in capturing people’s real-life experiences of their partners and their correlates with blood pressure. Assigning partners to be autonomy supportive in a laboratory interaction would likely not override years of less supportive experiences with partners. Thus, the mixed methods employed in this research aimed to capture directionality of effects without sacrificing ecological validity. However, and particularly in examining the long-term impact of autonomy support, effect sizes for autonomy support were small to moderate, ranging from *d* = .34 to .45 for the longitudinal associations to *d* = .69 for in-lab effects on blood pressure. In all likelihood, a myriad of other health and psychological predictors affect diastolic blood pressure. Nevertheless, these consistent, albeit modest, relations across the two methodological approaches supported our basic assertion that autonomy support plays an important role in influencing blood pressure.

Across all studies, autonomy support was linked to diastolic but not systolic blood pressure. These results (and null effects) were consistent in both studies for these relatively young adult samples. Psychosocial stress has powerful effects on diastolic blood pressure in particular (Rainforth et al. 2007; Uchino et al. 1996), which is the predominant indicator of physical health in adults and young adults (Franklin et al. 2001; Kannel et al. 1971), while systolic blood pressure is a better indicator in older adults (Franklin et al. 2001).

## Limitations and future directions

There are limitations to this study that are important to highlight, especially because we consider this research connecting autonomy support to blood pressure to be preliminary but promising as a direction for future research. First, the experimental study was somewhat underpowered with approximately 20 participants per condition in each; despite this, the findings of this study, coupled with the well-powered longitudinal study, support the links between autonomy support and blood pressure. Nevertheless, though the two studies relied on very different methods, both were correlational and retrospective in nature and further experimental research is needed to allow causal conclusions. To this end, future studies could bring both partners into the lab to invoke conflict, and capture autonomy-supportive behaviors as they occur. Previous studies applying SDT have included observational studies of couple conflicts and observed autonomy support (e.g., Knee et al. 2005), but have not included blood pressure outcomes. Looking at the behavioral components of autonomy support most closely associated with blood pressure changes would be instructive.

Next, we focused on diastolic blood pressure, and did not obtain multiple indicators of cardiovascular health to triangulate these relations with autonomy-supportive romantic partners. We could imagine, for example, adding impedance cardiography to assess challenge and threat motivational states (Blascovich and Mendes 2000) during a stressful partner conflict task as an additional index of cardiovascular responding. Finally, Study 2 used a more robust measure of perceived autonomy support that included conditional regard—a negative indicator of autonomy support—but we did not measure conditional regard during conflict. Understanding the dynamics of conditional regard during conflict would be important for future research as these behaviors may predict rises in blood pressure above and beyond a lack of autonomy-supportive behaviors during conflict.

Future research might also investigate other potential mechanisms of why autonomy-supportive partners are beneficial to physical health. For example, stress and behavioral coping may also play a role: a number of studies indicate that in general, perceiving autonomy support from others is associated with reduced self-reported stress and more effective ways of coping with stress. Research in the workplace has shown that job-related autonomy support is linked to less anxiety and self-reported stress at work (Karasek and Theorell 1990), and lower frustration when carrying out important tasks at work (De Cuyper and De Witte 2006; Spector and Jex 1991; Parker and Decotiis 1983). Additionally, perceiving autonomy support from others helps individuals to experience a greater sense of energy or vitality, and lower depression and burnout, all of which are important indicators of physical health and potential buffers to the negative effects of stressors (Gagné et al. 2003; Lynch et al. 2005; Ryan and Frederick 1997; Reis et al. 2000). These and other mechanisms are important to explore as they point to potential points of intervention to improve blood pressure within a romantic dyad. Similarly, it would be informative to compare autonomy support with other well-researched forms of support (e.g., invisible support; Bolger et al. 2000), to examine unique, overlapping, or interactive links with autonomy support on blood pressure.

In sum, this research extends the body of literature attesting to the myriad of wellness benefits of autonomy support in close relationships, and romantic relationships specifically, by revealing benefits to blood pressure. That autonomy-supportive romantic partners may not only protect against raising blood pressure, but may actually lower blood pressure, suggests potentially far-reaching effects of close others on physical health. This research also carries implications for health care interventions and couples therapy. For example Dunbar and colleagues (in press) recently showed that an intervention that enhanced autonomy support provided by spousal and partner caregivers predicted healthier and more rapid changes in patients’ diets after cardiovascular incidents. Working with couples to increase the autonomy support they provide each other, especially during times of conflict, appears to not only improve relationship functioning, but may also have positive effects on physical health.

## Footnotes

1 Blood pressure measurements involved a short interaction with the experimenter and were taken before and after the stress activity to avoid distracting participants. Blood pressure recovery is lasting, and recovery measurements can reliably be taken up to an hour after the intervention (e.g., Steptoe et al. 2006).

2 One participant was removed from analyses because of a blood pressure measurement malfunction: the participant’s measurements at Time 2a and Time 2b (at the end of the study) were 124 and 58 respectively, a difference of 66 units over a 2-min period. This participant was clearly an outlier as the next closest difference between these two time-points was 12 points, and the average difference was approximately 5 points.
